# Levofloxacin-Amoxicillin/Clavulanate-Rabeprazole versus a Standard Seven-Day Triple Therapy for Eradication of *Helicobacter pylori* Infection

**DOI:** 10.1155/2014/158520

**Published:** 2014-06-05

**Authors:** Ming-Cheh Chen, Wei-Yi Lei, Jen-Shung Lin, Chih-Hsun Yi, Deng-Chyang Wu, Chi-Tan Hu

**Affiliations:** ^1^Division of Hepatology and Gastroenterology, Department of Internal Medicine, Lotung Poh-Ai Hospital, Lo-Hsu Foundation, No. 83, Nanchang Street, Luodong, Yilan 265, Taiwan; ^2^Division of Gastroenterology, Department of Internal Medicine, Hualien Tzu-Chi Hospital, Buddhist Tzu-Chi Medical Foundation, No. 707, Section 3, ChungYang Road, Hualien 970, Taiwan; ^3^Research Center of Hepatology, Hualien Tzu-Chi Hospital, Buddhist Tzu-Chi Medical Foundation, No. 707, Section 3, ChungYang Road, Hualien 970, Taiwan; ^4^Department of Internal Medicine, School of Medicine, College of Medicine, Tzu-Chi University, No. 701, Section 3, ChungYang Road, Hualien 970, Taiwan; ^5^Division of Gastroenterology, Department of Internal Medicine, Kaohsiung Medical University Hospital, No. 100, TzYou 1st Road, Kaohsiung 807, Taiwan; ^6^Department of Internal Medicine, School of Medicine, College of Medicine, Kaohsiung Medical University, No. 100, Shih-Chuan 1st Road, Kaohsiung 807, Taiwan

## Abstract

The resistance rates of *Helicobacter pylori* to amoxicillin and metronidazole therapy are higher in eastern Taiwan as compared to national and worldwide rates. The high resistance rate in this territory justified a search for a better eradication regimen. We conducted an open-labeled, prospective, randomized, and controlled study in a tertiary referral hospital in eastern Taiwan. Between December 2007 and December 2009, a total of 153 *Helicobacter pylori*-positive, therapy-naïve patients with a positive rapid urease test were recruited for random assignment to two seven-day treatment groups: levofloxacin (500 mg), amoxicillin/clavulanate (875 mg/125 mg), and rabeprazole (20 mg) twice per day (LAcR) or clarithyromicin (500 mg), amoxicillin (1000 mg), and rabeprazole (20 mg) twice per day (CAR). *Helicobacter pylori* eradication was assessed using the ^13^C-urea breath test or rapid urease test performed at least 4 weeks after the end of treatment. After exclusion, 146 patients were enrolled and allocated in the study. The *Helicobacter pylori* eradication rates analyzed by both intention to treat (78.1% versus 57.5%, *P* = 0.008) and perprotocol (80.9% versus 61.8%, *P* = 0.014) were significantly higher for the LAcR group. In conclusion, the seven-day LAcR regimen provided improved *Helicobacter pylori* eradication efficacy when compared with the standard CAR triple therapy in eastern Taiwan.

## 1. Introduction

*Helicobacter pylori* (*H. pylori*) colonizes the human stomach chronically and is the causative agent of numerous benign and malignant gastric diseases [1]. A seroprevalence study in Taiwan showed that the seropositivity of* H. pylori* infection was 54.4% [2]. Eradication of* H. pylori* with a standard triple therapy using a proton pump inhibitor (PPI), amoxicillin, and clarithromycin or metronidazole was recommended to prevent gastric cancer and to avoid recurrence of peptic ulcer diseases [1, 3]. The recommended duration of the standard triple therapy from Asia-Pacific Consensus Guidelines is seven days [4]. Clarithromycin or metronidazole is suggested if the* H. pylori* local primary resistance rate is lower than 15~20% for the former or less than 40% for the latter [3]. Antimicrobial resistances in many countries have increased. As a consequence, the triple therapy eradication rate was less than 80% on an intention-to-treat (ITT) basis [5]. In eastern Taiwan, the primary resistance rates of metronidazole (51.9%), amoxicillin (36.1%), and clarithromycin (13.5%) in clinical isolates of* H. pylori* were higher [6] than those reported from other regions of Taiwan (Table 1) and worldwide [7–12]. However, the eradication rate of a seven-day triple therapy using clarithromycin and amoxicillin has never been reported for patients in eastern Taiwan.

Regimens containing levofloxacin (500 mg b.i.d.) plus amoxicillin (1 g b.i.d.) (LA) and a PPI have been evaluated recently as an alternative to the standard antibiotics [1, 13]. The use of LA-based regimens as a first-line treatment for* H. pylori* is encouraging but still controversial [11]. Beta-lactamase production in* H. pylori* is the principal mechanism of amoxicillin resistance [14]. In vitro studies [15, 16] and clinical trials [17–19] showed promising results by using amoxicillin plus a beta-lactamase inhibitor like clavulanic acid to attenuate* H. pylori* resistance to amoxicillin. Thus, our aim was to evaluate the efficacy and tolerability of a seven-day levofloxacin (500 mg b.i.d.), amoxicillin/clavulanate (1 g b.i.d.) plus rabeprazole (20 mg b.i.d) (LAcR) regimen versus the guideline-recommended seven-day triple therapy for treatment of* H. pylori* infection [4].

## 2. Methods

### 2.1. Study Population

In this single center prospective study, we included* H. pylori*-positive adult patients assessed by the rapid urease test between December 2007 and December 2009. We excluded patients under the age of 20, those who had received anti-*H. pylori* therapy previously, those with concomitant illness or conditions (i.e., cardiopulmonary, hepatic, renal, or neoplastic diseases), those who were pregnant or breast-feeding women, and those allergic to any of the drugs used. The protocol was approved by the Institutional Review Board (IRB) of Buddhist Tzu-Chi General Hospital (IRB 096-28) and registered in ClinicalTrials.gov (NCT01575899). Informed consents were obtained from all participants.

### 2.2. Study Design

Eligible patients were assigned into two groups by a computer generated random table with blocks based on gender. Sealed envelopes which were opened in the outpatient clinic without blinding were used for allocation concealment. The LAcR group received levofloxacin, 500 mg (Cravit, Daiichi-Sankyo, Japan) b.i.d., amoxicillin/clavulanate, 875 mg/125 mg (Augmentin, GlaxoSmithKline, UK) b.i.d., and rabeprazole, 20 mg (Pariet, Eisai, Japan) b.i.d., for 7 days. The standard triple therapy group served as the control group and was treated with clarithyromicin, 500 mg (Klaricid, Abbott, USA) b.i.d., amoxicillin, 1000 mg (Amoxicillin capsule 250 mg, Yung-Shin, Taiwan) b.i.d., and rabeprazole, 20 mg b.i.d. (CAR), for seven days.

### 2.3. Drug Compliance and Adverse Events

Drug compliance was defined as intake of more than 80% of each prescribed medication. Compliance and incidence of adverse events were collected by phone calls and in outpatient clinics. Each symptom was graded as either absent or present.* H. pylori* eradication was established based on a negative ^13^C-urea breath test or a negative rapid urease (CLO) test. The confirmation tests were carried out at least 4 weeks after completion of eradication therapy by operators unaware of the patients' medication and* H. pylori* status.

### 2.4. Statistical Analysis

The primary end point of this study was to evaluate the eradication rate of the LAcR regimen. The evaluation of* H. pylori* eradication efficacy was performed on both an intention-to-treat (ITT) and a perprotocol (PP) analyses. The sample size was predetermined for this paired cohort study, taking the following parameters into consideration: initial estimate of the difference in efficacy = 15% (i.e., 87% versus 72%) [5, 13]; alpha = 0.05; power = 80%, and gamma = 0.2. The number of patients thus calculated in each group was 88. The final sample size was 100 patients when assuming that 15% of the patients were lost to follow-up. Interim analyses with periodic reports every 4 months were requested by our IRB, which allowed investigators to adjust study course according to the interim results.

Categorical data were compared using the chi-square test employing Yates correction for continuity or Fisher's exact test as appropriate. Continuous data were compared with Student's *t*-test and expressed as mean ± SD. The stratified chi-square test was used for subgroup analysis between the two groups. Multiple logistic regression analyses were used for determination of major factors that affected treatment efficacy. Statistical analyses were performed using the SPSS 12.0 statistical software for Windows.

## 3. Results

### 3.1. Interim Analysis Result

The fifth interim analysis performed on December 2009 revealed that the difference in the eradication rates between the LAcR and CAR regimens was statistically significant (*P* = 0.014). According to the stopping boundaries corresponding to the fifth interim analysis for the Pocock (0.0158) or O'Brien-Fleming approach (0.0417) [20], this study was terminated early for ethical reasons. A total of 153 cases were screened, 7 cases were excluded, and 146 cases were enrolled and treated.

### 3.2. Patient Characteristics

**Figure 1:**
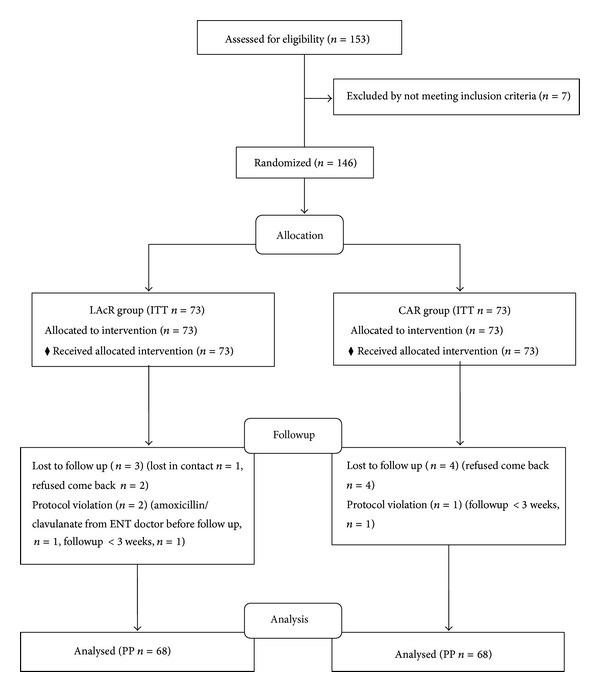
The participant flow chart.

The participant flow for this study is shown in Figure 1. As shown in Table 2, the baseline demographic data including age, gender, area of residence, endoscopic diagnosis, and follow-up methods were not significantly different between the two groups. The area of residence was defined by the domicile addresses registered by the participants. An address was considered “urban” if located in a city, while it was considered “rural” if in a township. Old population in this study, according to the current definition of the United Nations, was defined as ≥60 years old.

### 3.3. Effects of Therapy on* H. pylori* Eradication

The PP eradication rates (PP-ER) for the LAcR and the CAR groups were 55/68 (80.9%) and 42/68 (61.8%), respectively (*P* = 0.014). The ITT eradication rates (ITT-ER) for the LAcR and the CAR groups were 57/73 (78.1%) and 42/73 (57.5%), respectively (*P* = 0.008) (Table 3). In the subgroup analysis, compared with the CAR regimen, the LAcR therapy showed significantly higher PP-ER (52.6% versus 83.9%, *P* = 0.006) and ITT-ER (51.3% versus 77.1%, *P* = 0.021) in patients ≧54 years old, higher PP-ER (55.3% versus 82.9%, *P* = 0.006) and ITT-ER (53.1% versus 81.4%, *P* = 0.004) in those living in rural areas, and higher PP-ER (57.1% versus 84.6%, *P* = 0.009) and ITT-ER (52.6% versus 81.4%, *P* = 0.006) in those without endoscopic diagnosis of peptic ulcer diseases (Table 3). A multivariate logistic regression analysis confirmed that the unique factor that led to successful* H. pylori* eradication was the modality of treatment (Table 4).

### 3.4. Tolerance to* H. pylori* Eradication Therapy

One patient from the LAcR group developed severe vomiting during the therapy. This patient received supportive treatment at the emergency room. After recovery, she stopped the LAcR regimen and was lost to follow-up. The association between this event and the LAcR therapy could not be confirmed. Mild adverse events were reported by a few patients in both groups without significant differences (Table 5).

## 4. Discussion

Our study results suggest that the standard seven-day triple therapy is not suitable for patients in Eastern Taiwan because the* H. pylori* ITT-ER was only 57.5%. The LAcR regimen achieved a significantly higher* H. pylori* eradication rate, suggesting that the use of amoxicillin/clavulanate can increase the overall response rate. In addition, the LAcR regimen may have attenuated the influence of some socioeconomic factors contributing to a high failure rate by the standard triple therapy among rural residents. In fact, we found that the differences on primary resistance rates of metronidazole (52.8% versus 47.7%, *P* = 0.95), amoxicillin (22.2% versus 25.0%, *P* = 0.98), and clarithromycin (13.9% versus 22.7%, *P* = 0.58) in clinical isolates of* H. pylori* from patient living in urban and rural areas of eastern Taiwan were not statistically significant [21].

The efficacy of this seven-day levofloxacin plus amoxicillin/clavulanate (LAc) based regimen has never been reported previously. However, we found a similar study in northern Taiwan that evaluated a seven-day regimen using LA as a first-line therapy. As expected, this “LA” combination revealed an ITT-ER of 74.2%, which is inferior to our “LAc” combination with an ITT-ER of 78.1% [11].

Two studies evaluated the antiresistance effect of amoxicillin/clavulanate to its counterpart, amoxicillin, in a seven-day PPI, clarithromycin plus amoxicillin/clavulanate or amoxicillin regimen. One (with omeprazole) showed a significant improvement in the ITT-ER (86.6% versus 66.6%, *P* < 0.05) [17]. The second study (with esomeprazole) showed a positive trend but did not achieve statistical significance (ITT-ER 72% versus 62%, *P* = 0.2188) [18]. Similarly, a trial comparing omeprazole, azithromycin plus amoxicillin/clavulanate, or amoxicillin also showed a beneficial trend (ITT-ER 86% versus 82%, *P* = 0.801; PP-ER 91.5% versus 85.4%, *P* = 0.543) but did not attain statistical significance [19]. In two studies, the omeprazole, metronidazole plus amoxicillin/clavulanate regimen for 2 weeks provided an 80.5% PP-ER in children from Iran [22] and a 76.4% PP-ER in adults from China [23].

Other strategies to increase the eradication rate when confronted with antibiotic resistance are to increase dosage and to extend treatment duration. Better* H. pylori* eradication rates have also been achieved elsewhere by 10- to 14-day versus seven-day LA regimens [24–28] with only two studies having less than 80% ITT-ER [24, 29]. We hypothesize that if we extend the duration of the LAcR regimen to 10–14 days, we will anticipate an ITT-ER beyond 80%. The optimal dosage and duration of a LAc-based regimen for an area with high* H. pylori* resistance to amoxicillin and/or clarithromycin require more investigations.

In this study, we chose rabeprazole as the PPI because its metabolism is less affected by CYP2C19 [30, 31] and CYP3A4 genotypes [31]. As a result, we anticipated less interpersonal variation in drug response, but this strategy may lead to a possible reduction in the synergistic effect of a PPI with antibiotics like clarithromycin [32, 33], metronidazole [33], or fluoroquinolone [34]. A study in China reported an ITT-ER of 75.4% for a seven-day LA-10 mg rabeprazole regimen, which was inferior to those of a seven-day LA-20 mg esomeprazole (85.2%) and a seven-day LA-40 mg esomeprazole (87.1%) regimen, respectively [35]. Thus, the susceptibility variation of CYP2C19/CYP3A4 genotypes and types/dosage of various PPIs used in this LAc-based regimen are also important considerations to improve* H. pylori* eradication rate. We have been engaged in an investigation of the influence of CYP2C19 genotypes on the efficacy of* H. pylori* eradication by the LAcR regimen as a second-line treatment.

We found that the* H. pylori* eradication rate of this LAcR regimen was not affected by common host factors (i.e., old age, rural residence, and ulcer/nonulcer status) that could affect the standard triple therapy. The poorer* H. pylori* eradication rate by the standard triple therapy in rural area may not be true in different countries. For example, rural areas in northern Wales had a higher ITT-ER (92%) by the standard triple therapy [36] when compared to rural China (PP-ER 69.59%, ITT-ER 65.6%) [37]. Our study results first point out that* H. pylori* eradication rate could be affected by problems in rural areas such as poor sanitary conditions and personal hygiene, antibiotic abuse in agriculture, limited education resources, and inadequate access to clean water. Since the majority of previous clinical trials on* H. pylori* treatment were performed in large city hospitals, the influence of rural residence on* H. pylori* eradication rate needs more therapeutic trials to confirm. Above all, one of the advantages of the LAcR regimen for first-line use is that clinicians need not be concerned about patients' socioeconomic status before prescribing this regimen in an area with high* H. pylori* resistance rate to conventional antibiotics. Their concerns can focus on penicillin allergy, compliance, and possible side effects. The limitation of this study is that the local* H. pylori* resistance rates to levofloxacin and amoxicillin/clavulanate are unknown.

## 5. Conclusion

The seven-day LAcR regimen for* H. pylori* eradication is a viable alternative to the standard seven-day clarithromycin-based triple therapy for eastern Taiwan. It is a rescue regimen before the discovery of an optimal first-line therapy for a region with high ampicillin and/or clarithromycin resistance rates. The efficacy of the standard eradication therapy is probably reduced by the high* H. pylori* resistance rate to amoxicillin and/or clarithromycin.

## Figures and Tables

**Table 1:** Primary resistance rate of *H. pylori* reported in Taiwan (published since 2000 to 2010 D.C.)

Author (study period) [reference]	Location (region)	Metronidazole	Clarithromycin	Amoxicillin	Levofloxacin	Tetracycline
Hu et al. (2004~2005) [6]	Hualien (east)	51.9%	13.5%	36.1%	Nil	0%
Yang et al. (1997~1999) [7]	Taipei (north)	9% (6/67)	18% (12/67)	Nil	Nil	Nil
Poon et al. (1998~2000) [8]	Taichung (west)	41.7% (35/84)	10.7% (9/84)	0% (0/84)	Nil	Nil
Poon et al. (2001~2004) [8]	Taichung (west)	25.4% (34/134)	6.7% (9/134)	0% (0/134)	Nil	Nil
Hung et al. (1998~2007) [9]	Tainan (south)	27.6% (58/210)	9.5% (20/210)	1.0% (2/210)	5.7% (12/210)	0.5% (1/210)
Wu et al. (2007~2008) [10]	Kaohsiung (south)	33.5% (56/167)	6.6% (11/167)	0.6% (1/167)	10.2% (17/167)	0.6% (1/167)
Liou et al. (2007~2009) [11]	Taipei (north)	Nil	7.5% (20/266)	2.5% (7/279)	5.7% (16/280)	Nil

**Table 2:** Demographic characteristics of participants.

	All (*n* = 146)	LAcR (*n* = 73)	CAR (*n* = 73)	*P* value
Mean age (years)	53.73 ± 13.29	52.82 ± 12.08	54.63 ± 14.42	0.413
Age < 60 years old, *n* (%)	98 (67.1)	51 (69.9)	47 (64.4)	
Age ≧ 60 years old, *n* (%)	48 (32.9)	22 (30.1)	26 (35.6)	0.481
Gender				
Male, *n* (%)	71 (48.6)	38 (52.1)	33 (45.2)	
Female, *n* (%)	75 (51.4)	35 (47.9)	40 (54.8)	0.408
Resident area				
Urban, *n* (%)	54 (37.0)	30 (41.1)	24 (32.9)	
Rural, *n* (%)	92 (63.0)	43 (58.9)	49 (67.1)	0.304
Endoscopic finding				
With peptic ulcer, *n* (%)	65 (44.5)	30 (41.1)	35 (47.9)	
Without peptic ulcer, *n* (%)	81 (55.5)	43 (58.9)	38 (52.1)	0.405
Follow-up method				
C13 urea breath test	130 (89.0)	65 (89.0)	65 (89.0)	0.881
CLO test	9 (6.2)	5 (6.9)	4 (5.5)	
Lost to follow up	7 (4.8)	3 (4.1)	4 (5.5)	

**Table 3:** Comparison of eradication rate and subgroup analysis.

	Perprotocol analysis				Intention-to-treat analysis			
	LAcR, *n* (%)	CAR, *n* (%)	*P* value	Odd ratio (95% CI)	LAcR, *n* (%)	CAR, *n* (%)	*P* value	Odd ratio (95% CI)
All	55 (80.9)	42 (61.8)	0.014*	2.619 (1.20~5.70)	57 (78.1)	42 (57.5)	0.008*	2.629 (1.28~5.42)
Age								
<60 years old	37 (75.5)	31 (72.1)	0.710	1.194 (0.47~3.03)	37 (72.5)	31 (66.0)	0.479	1.364 (0.58~3.23)
≧60 years old	20 (95.2)	11 (42.3)	0.000*	27.273 (3.17~235)	20 (90.9)	11 (42.3)	0.000*	13.636 (2.62~70.91)
Gender								
Male	30 (81.1)	20 (64.5)	0.123	2.357 (0.78~7.11)	30 (78.9)	20 (60.6)	0.091	2.438 (0.86~6.94)
Female	25 (80.6)	22 (59.5)	0.060	2.841 (0.94~8.59)	27 (77.1)	22 (55.0)	0.044*	2.761 (1.01~7.55)
Resident area								
Urban	21 (77.8)	16 (76.2)	0.748	1.094 (0.28~4.23)	22 (73.3)	16 (66.7)	0.594	1.375 (0.43~4.44)
Rural	34 (82.9)	26 (55.3)	0.006*	3.923 (1.45~10.62)	35 (81.4)	26 (53.1)	0.004*	3.870 (1.50~10.02)
Endoscopic finding								
With peptic ulcer	22 (75.9)	22 (66.7)	0.426	1.571 (0.51~4.80)	22 (73.3)	22 (62.9)	0.368	1.625 (0.56~4.69)
Without peptic ulcer	33 (84.6)	20 (57.1)	0.009*	4.125 (1.38~12.36)	35 (81.4)	20 (52.6)	0.006*	3.938 (1.45~10.68)

*P < 0.05.

**Table 4:** Multiple logistic regression analysis.

	Perprotocol		Intention to treat	
	Odds ratio (95% CI)	*P* value	Odds ratio (95% CI)	*P* value
Age				
<60 years old	1.0 (referent)		1.0 (referent)	
≥60 years old	1.33 (0.61–2.92)	0.478	1.16 (0.55–2.47)	0.696
Gender				
Male	1.0 (referent)		1.0 (referent)	
Female	1.14 (0.53–2.46)	0.737	1.18 (0.57–2.44)	0.648
Resident area				
Urban	1.0 (referent)		1.0 (referent)	
Rural	1.49 (0.65–3.42)	0.342	1.12 (0.53–2.37)	0.775
Endoscopic finding				
With peptic ulcer	1.0 (referent)		1.0 (referent)	
Without peptic ulcer	1.05 (0.48–2.26)	0.909	1.05 (0.51–2.16)	0.900
Treatment				
CAR	1.0 (referent)		1.0 (referent)	
LAcR	2.67 (1.22–5.84)	0.014*	2.57 (1.24–5.33)	0.011*

*P < 0.05.

**Table 5:** Comparison of side effects.

	LAcR^a^	CAR	*P* value
All, *n* (%)	10 (13.7)	11 (15.1)	0.814
Abdominal pain	2	2	
Flatus/abdominal fullness	1	3	
Loose stool/diarrhea	3	1	
Nausea/hiccough	4	4	
Vomiting	2^a^	0	
Change in appetite	2	4	
Insomnia	1	0	

^a^One of the two cases stopped the LAcR therapy due to severe vomiting.
